# Beyond Longitudinal Sampling: Translating Sexually Transmissible Enteric Infection Research Into Gastrointestinal Diagnostic Pathways

**DOI:** 10.2196/102167

**Published:** 2026-10-02

**Authors:** Mohammed Abdulrasak, Harald Bagger-Jörgensen

**Affiliations:** 1 Department of Clinical Sciences, Lund University, Jan Waldenströms gata 14, plan 2, Malmö, Skåne, 205 02, Sweden, 46 765527936

**Keywords:** sexually transmitted disease, sexually transmitted enteric infection, gay, bisexual, men who have sex with men, longitudinal sampling, acceptability, feasibility

We read with interest the study by Fountain et al [1] assessing the acceptability and feasibility of longitudinal sampling for sexually transmissible enteric infections (STEIs) among gay, bisexual, and other men who have sex with men (GBMSM) [1]. The study highlights important gaps in our understanding of STEI carriage, persistence, and transmission dynamics.

The conventional divide between “sexually transmitted” and “enteric” pathogens is less clear-cut than has historically been presumed. Enteric pathogens were traditionally conceptualized as foodborne or fecal-orally transmitted organisms, yet sexual transmission within GBMSM networks is increasingly recognized. While organisms such as *Shigella* and *Campylobacter* are now well described in this setting [2], other pathogens, including *Salmonella* spp and *Entamoeba histolytica*, may also be encountered in association with sexual exposure and anorectal inflammatory syndromes [3,4].

The authors appropriately emphasize the public health importance of this issue, including antimicrobial resistance, asymptomatic carriage, and the need for better longitudinal epidemiological data. However, these observations also carry important implications for day-to-day gastrointestinal clinical practice. Patients with STEIs may present not only to sexual health services but also to emergency departments, primary care, and gastroenterology clinics. In such settings, the possibility of sexually transmissible enteric infection may not be immediately considered, particularly when symptoms resemble inflammatory bowel disease.

This overlap is clinically important. Sexually transmitted colorectal infections may mimic inflammatory bowel disease, potentially resulting in diagnostic delay, unnecessary colonoscopy, inappropriate corticosteroid or biologics exposure, and missed opportunities for transmission control [5]. This is particularly relevant in patients presenting with proctitis or proctocolitis, where standard clinical, endoscopic, and radiological features may not reliably distinguish inflammatory bowel disease from infectious causes.

Building on the work of Fountain et al [1], we suggest that future clinical pathways should translate STEI research into a more structured diagnostic approach for GBMSM presenting with proctocolitis. Such an approach could follow 3 steps. First, perform initial rectal sexually transmissible infection testing after careful, nonjudgemental sexual history taking, including testing for classical anorectal sexually transmitted pathogens where clinically indicated. Second, perform expanded enteric testing if symptoms persist or initial sexually transmissible infection testing is negative, using fecal multiplex gastrointestinal molecular panels that include bacterial and parasitic pathogens. Third, perform colonoscopy, although this should be reserved for selected cases, particularly when microbiological investigations are negative, symptoms persist, inflammatory markers remain elevated, or there is ongoing concern regarding inflammatory bowel disease, malignancy, or another noninfectious pathology.

This is not intended to suggest indiscriminate testing in all patients. Rather, it reflects a risk-adapted and behavior-informed diagnostic model. In this sense, careful sexual history taking becomes part of precision medicine, allowing clinicians to tailor microbiological investigations and treatment decisions according to individual exposure patterns, epidemiological context, and clinical phenotype.

Earlier recognition of STEIs in gastrointestinal clinical settings may improve case detection, facilitate appropriate partner notification, support antimicrobial resistance surveillance, and help interrupt transmission within sexual networks. As the spectrum of sexually transmissible infections continues to evolve, diagnostic pathways must similarly adapt to recognize the growing overlap between sexual health, enteric infection, and gastrointestinal inflammation.

## Abbreviations

GBMSM: gay, bisexual, and other men who have sex with men
STEI: sexually transmissible enteric infection
